# Deciphering cell wall sensors enabling the construction of robust *P. pastoris* for single-cell protein production

**DOI:** 10.1186/s13068-023-02428-7

**Published:** 2023-11-17

**Authors:** Le Gao, Jiao Meng, Wuling Dai, Zhaokun Zhang, Haofan Dong, Qianqian Yuan, Wuyuan Zhang, Shuguang Liu, Xin Wu

**Affiliations:** 1 Tianjin Institute of Industrial Biotechnology, Chinese Academy of Sciences, National Technology Innovation Center of Synthetic Biology, No. 32, Xiqi Road, Tianjin Airport Economic Park, Tianjin, 300308 China; 2 Beijing Chasing future Biotechnology Co., Ltd, Beijing, China

**Keywords:** Single-cell protein, *Pichia pastoris*, Cell wall sensor, Signal perturbations, Space–time productivity

## Abstract

Single-cell protein (SCP) production in the methylotrophic yeast *Pichia pastoris* has the potential to achieve a sustainable protein supply. However, improving the methanol fermentation efficiency and reducing carbon loss has been a long-standing challenge with far-reaching scientific and practical implications. Here, comparative transcriptomics revealed that *PAS_0305*, a gene directly associated with cell wall thickness under methanol stress, can be used as a target for unlocking cell wall sensors. Intracellular trehalose accumulation confirmed that cell wall sensors were activated after knocking out *PAS_0305*, which resulted in increased cell wall permeability. Genome-wide signal perturbations were transduced through the HOG module and the CWI pathway, which was confirmed to connected by *Pbs2-Mkk*. As a consequence of CWI pathway activation, Δ*PAS_0305* elicited a rescue response of cell wall remodeling by increasing the β-1,3-glucan content and decreasing the chitin/mannose content. Remarkably, perturbations in global stress signals led to a fine-tuning of the metabolic network of Δ*PAS_0305*, resulting in a superior phenotype with highest crude protein and methanol conversion rate of 67.21% and 0.46 gDCW/g. Further genome-scale metabolic models were constructed to validate the experimental results, confirming that unlocking cell wall sensors resulted in maximized flux from methanol towards SCP and effectively addressing the issue of carbon loss in methanol fermentation. This work sheds new light on the potential of manipulating cellular signaling pathways to optimize metabolic networks and achieve exceptional phenotypic characteristics, providing new strategies for constructing versatile cell factories in *P. pastoris*.

**Supplementary Information:**

The online version contains supplementary material available at 10.1186/s13068-023-02428-7.

## Introduction

The growing world population along with a shift toward diets rich in animal protein has led to an increased consumption of animal products and an ever-increasing pressure on the food supply. Currently, the world consumes approximately 1.25 billion tons of meat and dairy products per year, which falls short of the projected global demand for animal-derived protein by 2050 [1]. While plant-based protein sources like beans are nutritionally valuable, they also require arable land and water, resources that are becoming increasingly scarce as the demand for protein grows [2]. However, the industrialization of alternative protein sources is predicated on new cost-effective avenues, ensuring both economic and environmental sustainability. One such avenue is the production of single-cell proteins (SCP) using yeast and algal cells. This approach holds promise for resolving issues of both food and feed supplies. SCP can be produced on a commercial scale, offering a viable alternative to traditional protein sources.

SCP production can utilize a variety of non-food raw materials, thereby reducing costs and enhancing sustainability. In particular, methanol has gained attention as a non-food one-carbon (C1) feedstock and a promising fermentation substrate due to its abundance, low cost, and high reduction potential [3]. Considerable breakthroughs were achieved in methanol synthesis via CO_2_ hydrogenation [4], opening an avenue for methanol to become a renewable raw material in the future. As the highly flexible methanol supply is derived from both fossil and renewable resources, methanol could serve as an alternative to sugars as a raw material for bio-based manufacturing.

*Pichia pastoris*, a representative methylotrophic eukaryote, is capable of using methanol as the sole carbon source for the synthesis of SCP and other products [5]. However, a mass balance analysis by Vanz et al. indicated that up to 80% of the methanol is converted into CO_2_ during the methanol fed-batch fermentation of *P. pastoris* [6]. Thus reduction of carbon loss and enhancement of carbon metabolism is essential for further improving the conversion of methanol into SCP. Some efforts have been made to regulate methanol metabolism, including peroxisomal compartmentalization of the methanol utilization pathway [7], balancing assimilation and dissimilation pathways to enhance methanol metabolism [8], as well as utilizing synthetic biology and genetic engineering to improve the methanol fermentation efficiency [9, 10]. However, *P. pastoris* is adapted to methanol utilization under ecological conditions, and uses a complex network to control methanol metabolism [11, 12]. Currently, there is limited knowledge on genetic mechanisms that can be used to generate desirable phenotypes via global perturbations in *P. pastoris*, beyond adaptive laboratory evolution [13]. In spite of significant engineering efforts, it remains challenging to improve the performance of *P. pastoris* by only altering one or several metabolic pathways. Therefore, although *P. pastoris* has been used to produce SCP from methanol on a laboratory scale for decades, improving the methanol conversion efficiency and reducing carbon loss remains a long-standing challenge [14, 15]. Therefore, there is a pressing need to explore novel strategies that comprehensively target and optimize the intricate network of methanol metabolism in *P. pastoris*.

Here, activation of cell wall sensors was used as an effective breeding strategy for improving the methylotrophic performance of *P. pastoris.* Genome-scale signal perturbations were triggered by activating the cell wall sensors, resulting in maximal flux distribution from methanol towards biomass in *P. pastoris.* Extensive investigations have successfully unraveled the intricate underlying mechanism responsible for generating superior phenotypes in *P. pastoris* following activation of cell wall sensors. The global metabolism of *P. pastoris* was successfully reprogrammed for improved robustness during SCP overproduction, providing a new strategy for constructing versatile cell factories in *P. pastoris*.

## Results

### Screening of key target genes related to cell wall sensors by RNA-Seq

**Fig. 1:**
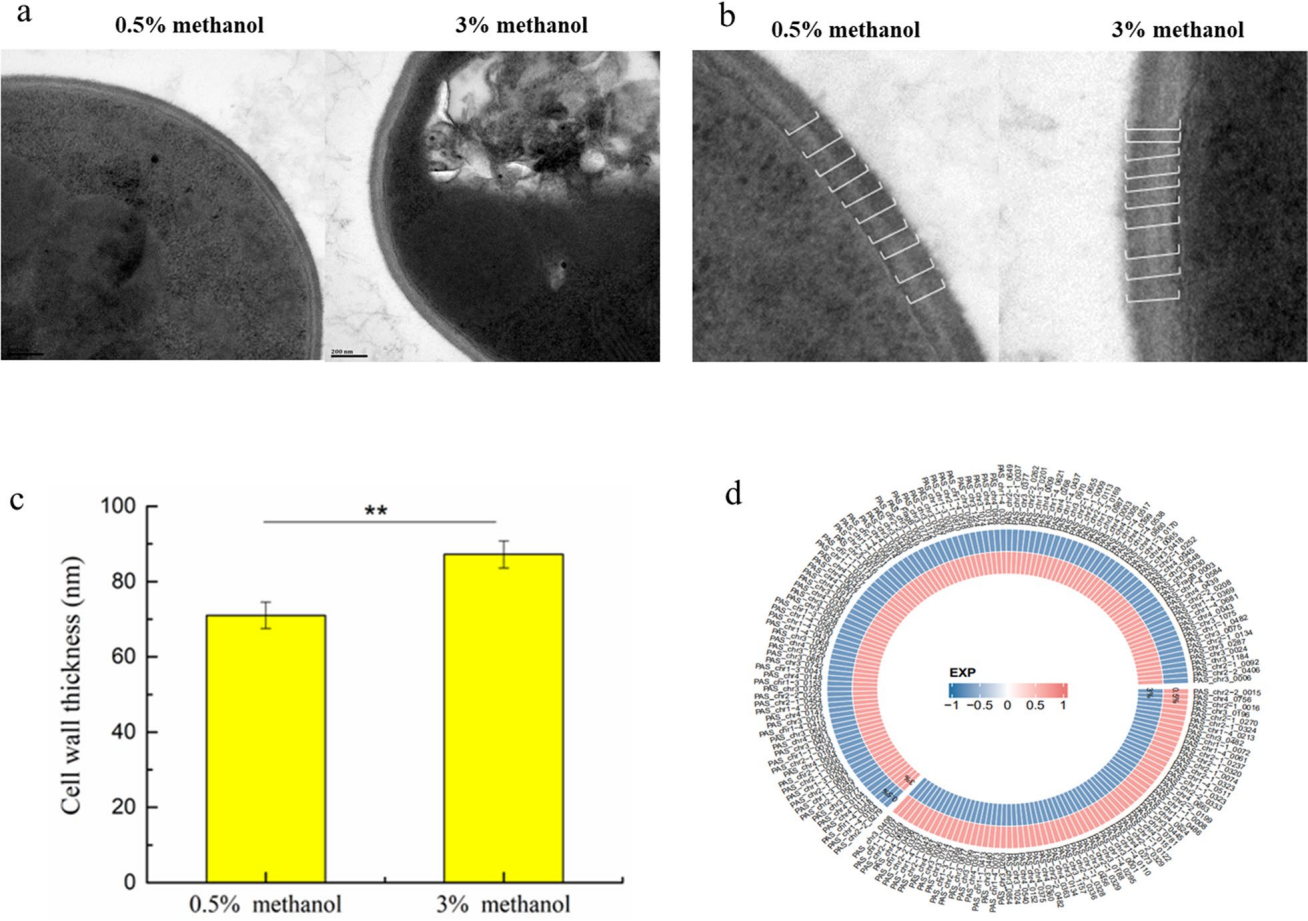
Transmission electron micrographs of whole cells (**a**), with a zoomed-in view for measurement of cell wall thickness (**b**), and average analysis of cell wall thickness (**c**) of *P. pastoris* cells grown under 0.5% and 3% methanol. **d** Hierarchical clustering of significantly differentially expressed genes in *P. pastoris X33* under 0.5% and 3% methanol

In this study, we found that the cell wall thickness of *P. pastoris* X33 increased with increasing methanol concentration (Fig. 1a, b). For example, the cell wall thickness of cells cultured in Delft basic salt medium containing 3% methanol was 22.8% greater than in the same medium with 0.5% methanol. To identify genes involved in cell wall synthesis in response to changes of methanol stress, RNA-Seq was conducted to compare gene expression changes between the cells cultured in media with different concentrations of methanol (Fig. 1c). The RNA-Seq data showed that 181 genes were significantly differentially expressed when the methanol concentration was increased from 0.5 to 3%, 115 of which were upregulated and 66 were downregulated. However, among these 181 differentially expressed genes, only 2 genes were related to cell wall synthesis, namely *PAS_chr4_0305* (*PAS_0305*) encoding an *O*-glycosylated protein required for cell wall stability, and *PAS_chr2-1_0454* (*PAS_0454*) encoding a major exo-1,3-beta-glucanase of the cell wall, involved in cell wall beta-glucan assembly (Additional file 1: Extended Data Table S1). The expression of *PAS_0305* and *PAS_0454* was upregulated by an average of 2.8- and 2-fold, respectively, with relative correlation with thickening of the cell wall in response to elevated methanol. Conversely, we hypothesized that deletion of *PAS_0305* and *PAS_0454* may alter the cell wall structure, thereby affecting the cell wall sensors. Therefore, we knocked out these two genes in wild-type *P. pastoris*. It is worth noting that there were no viable *PAS_0454* mutant transformants on the selective plates, indicating that *PAS_0454* is an essential gene for cell wall integrity of *P. pastoris*. However, the *PAS_0305* gene may participate in maintaining *P. pastoris* cell wall properties, unveiling the potential impact of cell wall sensors.

### Knockout of* PAS _0305 *increased the cell wall permeability of *P. pastoris*

**Fig. 2:**
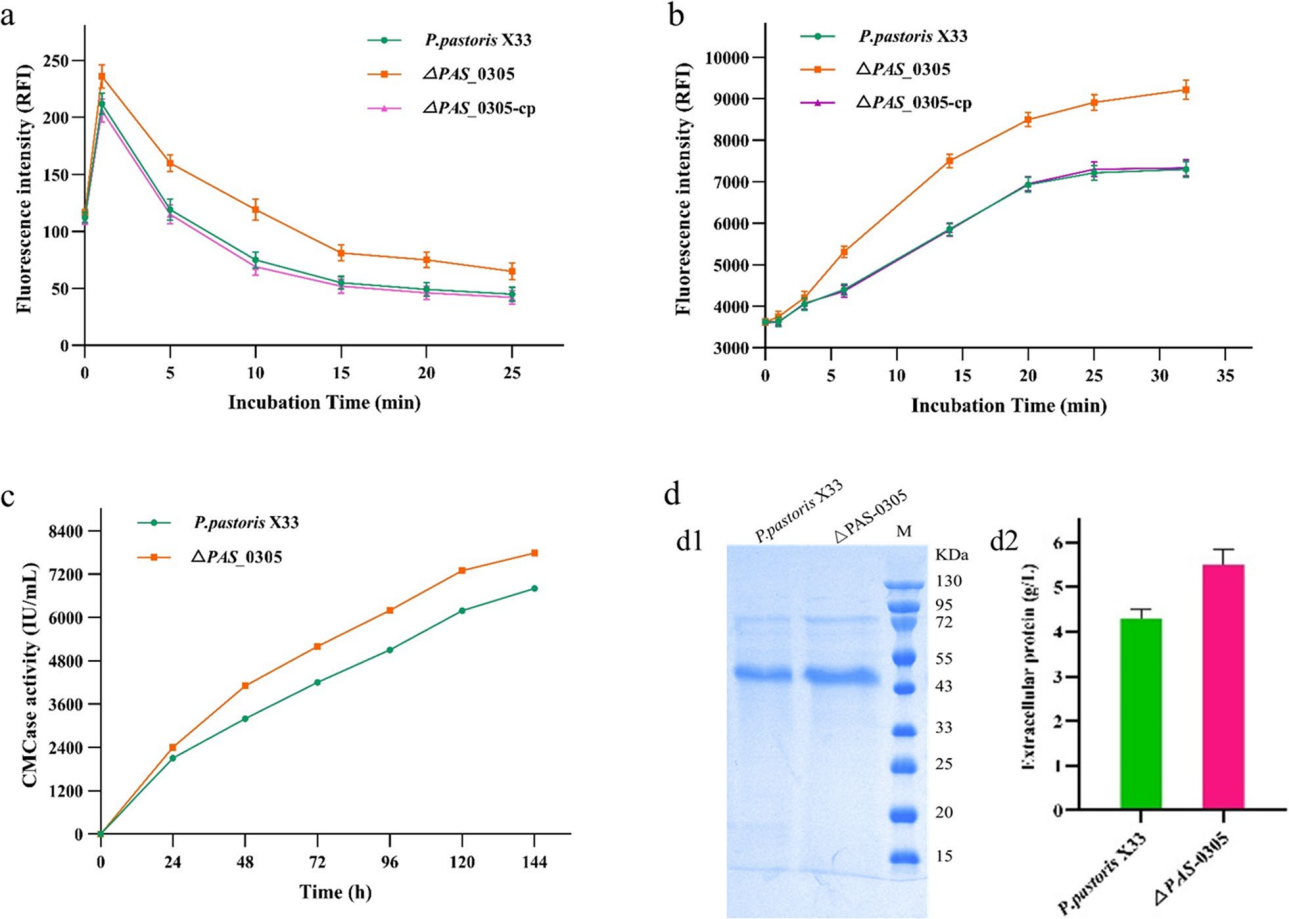
Analysis of changes in *P. pastoris* cell wall permeability after knocking out Δ*PAS-0305*. Assessment of whole-cell permeability via **a** intracellular Nile red fluorescence measurement; **b** intracellular propidium iodide fluorescence measurement; and **c** measurement of CMCase activity of heterologously HgCel5A. **d** Comparison of *P. pastoris* extracellular protein. d1, SDS-PAGE. d2, extracellular protein concentration analysis

The cell wall serves as the robust outermost layer of yeast cells, acting as the primary defense mechanism against external stresses and the initial sensor to perceive the surrounding environment [16]. Cell wall permeability plays an important role in the sensing of external stress by *P. pastoris.* To investigate cell permeability changes after knock out of *PAS_0305*, we used fluorescence spectroscopy to measure the whole-well accumulation of propidium iodide and Nile red, representative hydrophilic and hydrophobic compounds, respectively. The results showed that the accumulation rate and final levels of the two compounds were higher in the Δ*PAS_0305* strain than in the control strain, indicating an increase of cell wall permeability (Fig. 2a, b). This result was in agreement with a previous study, which found that the cell wall permeability of *Mycolicibacterium smegmatis* increased following alteration of SWU1gp39 [17].

Compared with the fluorescence spectroscopy analysis, measurement of the extracellular levels of a heterologously expressed protein provides a more straightforward and intuitive assessment of cell wall permeability. Hence, identical HgCel5A expression cassette with the P_AOX_ promoter and T_GAP_ terminator was introduced into both the Δ*PAS_0305* strain and the *P. pastoris* X33 strain at the same neutral PNSII-8 site using the Cas9 system. To further characterize HgCel5A secretion under identical growth conditions, fed-batch bioreactor cultivations were carried out. Throughout the entire induction period, both CMCase activity and protein secretion were superior in the Δ*PAS_0305*::*HgCel5A* strain compared to *P. pastoris* X33::*HgCel5A*. After 120 h, the extracellular protein levels and CMCase activity of Δ*PAS_0305*::*HgCel5A* reached a significantly higher level (Fig. 2c). During the fed-batch fermentation process, the extracellular protein concentration and CMCase activities of Δ*PAS_0305*::*HgCel5A* were 22.16% and 14.41% higher than those of *P. pastoris* X33::*HgCel5A*, at 5.50 vs. 4.28 and 7789 vs. 6808. IU/ml, respectively (Fig. 2c). The SDS-PAGE results further confirmed that knocking out *PAS_0305* promoted protein secretion by increasing cell wall permeability (Fig. 2d). This finding was consistent with the results obtained from fluorescence spectroscopy analysis.

**Fig. 3:**
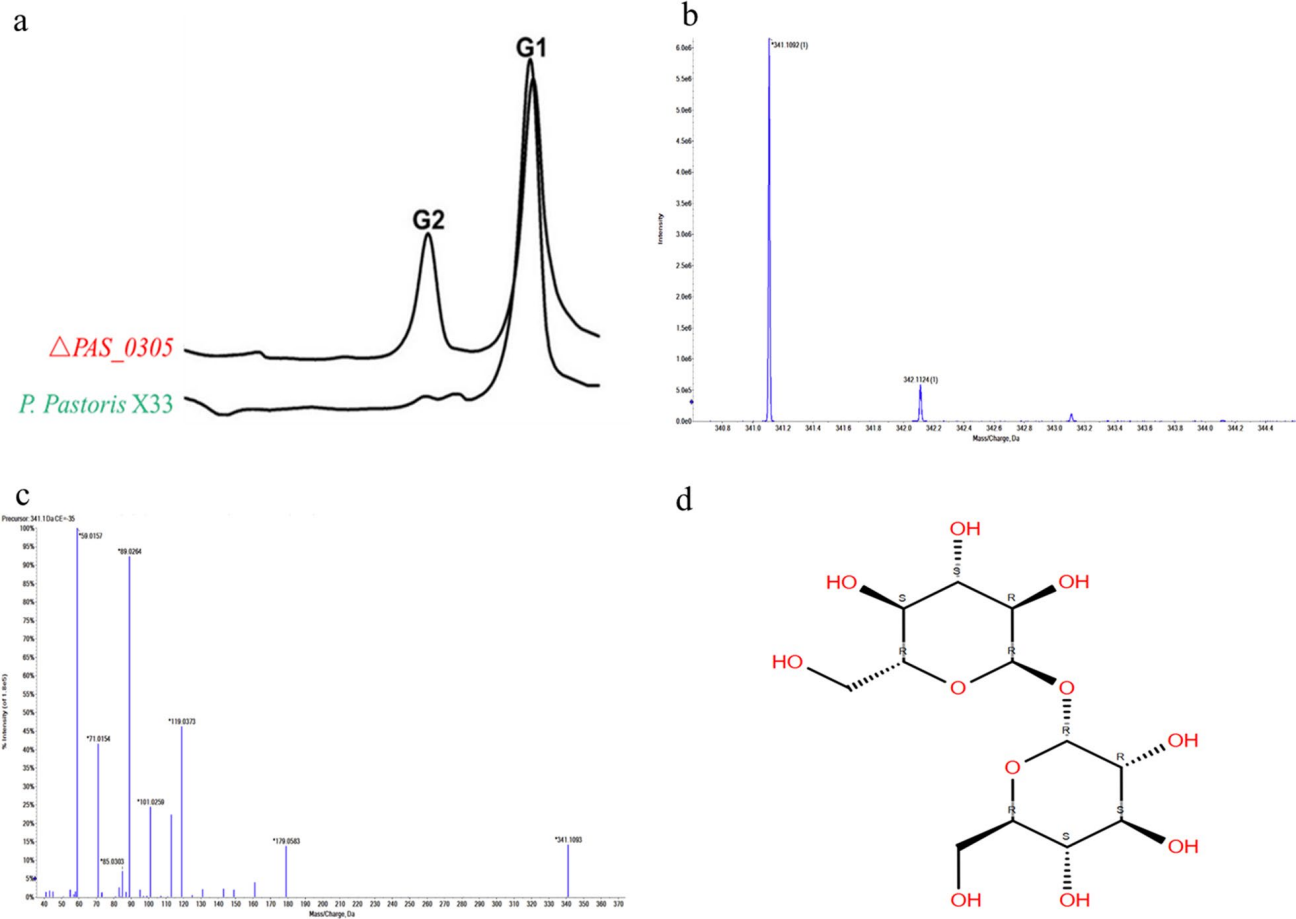
Analysis of intracellular osmolyte changes in Δ*PAS_0305*; **a** analysis of intracellular osmolytes via HPLC; **b** primary mass spectrum of the identified disaccharide. **c** Secondary mass spectrogram of the disaccharide. **d** Structure of the identified disaccharide

Further analysis confirmed that the Δ*PAS_0305* strain exhibited significantly higher accumulation of intracellular osmolytes, particularly disaccharides, compared to *P. pastoris* X33 (Fig. 3a). LC–MSMS analysis further identified the accumulated disaccharide as trehalose (Fig. 3b–d). Previous studies have demonstrated that the expression of genes involved in trehalose metabolism is rapidly upregulated in response to various stressors, such as heat, resulting in trehalose accumulation in *Saccharomyces cerevisiae* [18, 19]. In comparison to *P. pastoris* X33, the Δ*PAS_0305* strain exhibited a 32.71% increase of intracellular trehalose accumulation. This observation suggests that a cell wall sensor was activated by the increased cell wall permeability of *P. pastoris* after knockout of *PAS_0305*, triggering intracellular trehalose accumulation as a response to a perceived environmental stress (Fig. 3).

### Enhanced environmental tolerance of *P. pastoris* after activating cell wall sensors

**Fig. 4:**
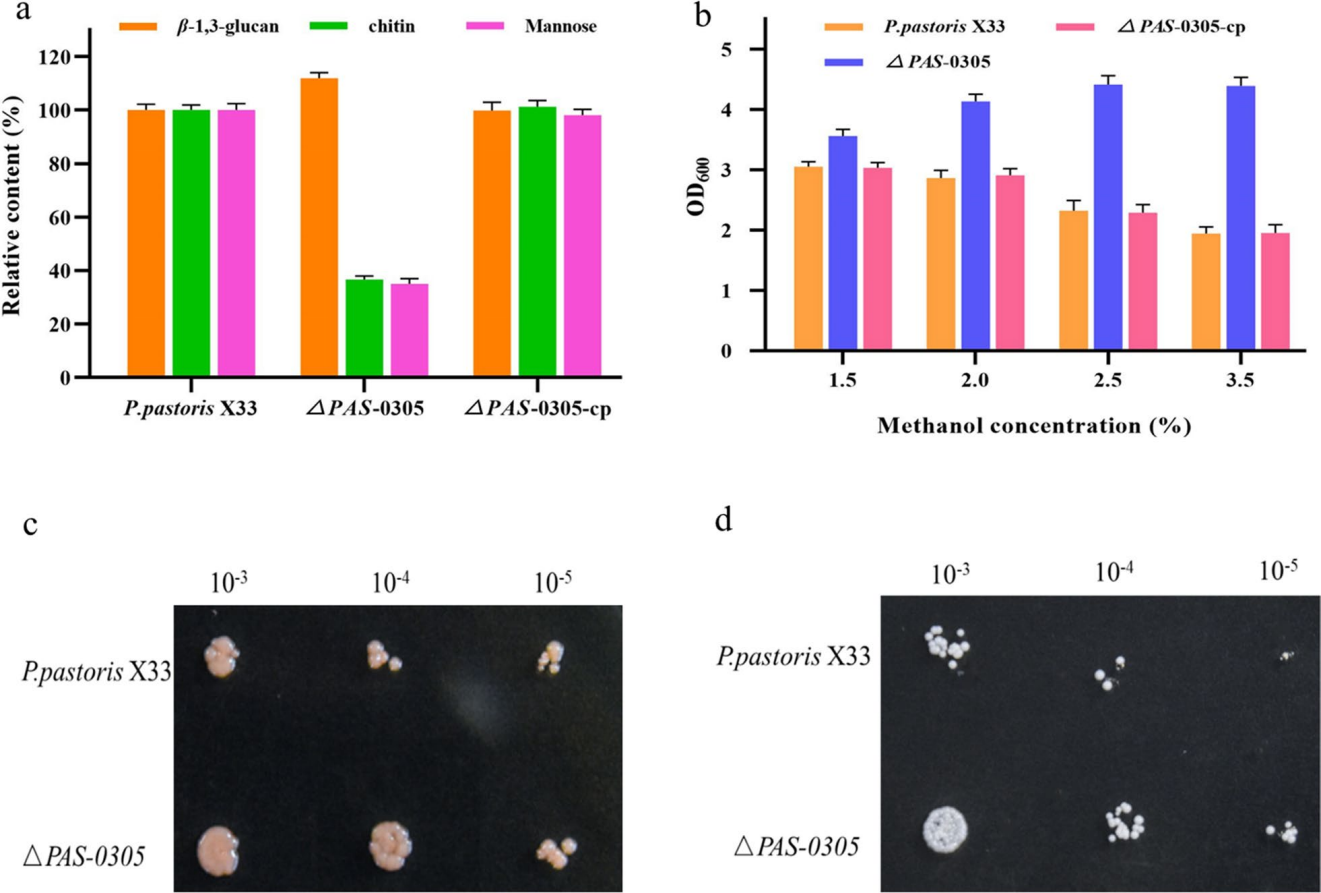
Comparison of the methylotrophic performance and industrial robustness between *P. pastoris X33* and Δ*PAS-0305*. **a** Analysis of cell wall composition. **b** Growth with different concentrations of methanol. **c** Sensitivity of strains to Congo red in YPD medium. **d** Sensitivity of strains to sodium dodecyl sulfate (SDS) in YPD medium

To investigate the impact of the *PAS_0305* gene knockout on cell wall composition, we compared Δ*PAS_0305* to its parental strain *P. pastoris* X33. A slight increase in lipid content was observed in the *PAS_0305* strain (7.21%) compared to the control strain (6.50%) (*P* < 0.005) (Table 1). Notably, the cell wall of the Δ*PAS_0305* strain exhibited significant decreases of 63.34 and 65.00% in chitin and mannose content, respectively. Conversely, the β-1,3-glucan content of the Δ*PAS_0305* strain was 11.97% higher than that of the control strain(*P* < 0.005) (Fig. 4a). These findings suggest that the cell wall composition of *P. pastoris* undergoes remodeling following the disruption of the *PAS_0305* gene. This observation is in agreement with previous research showing that overexpression of GPI-modifying cell wall proteins increased the β-1,3-glucan content, which enhances the resistance of *P. pastoris* X33 [20]. Additionally, the disruption of genes involved in chitin synthesis was found to increase cell wall resistance to CW, as chitin serves as the primary binding target for Congo red (CR) in yeast [21]. Ultimately, the increased β-1,3-glucan content and reduced chitin content in the *P. pastoris* cell wall contributed to its enhanced resistance to various environmental stresses.

**Table 1:** The methanol utilization rate and carbon loss parameters calculated using the iMT1026 v3.0 GSMM with the macromolecular composition of cells grown on methanol used as input for defining the stoichiometric coefficients

	Parameter	*P. pastoris* X33	Δ*PAS_0305*
Input	Protein (%)	56.62	67.21
	Carbohydrates (%) (β-1,3glucan + chitin + mannose)	27.90 (14.62 + 4.31 + 8.97)	21.09 (16.37 + 1.58 + 3.14)
	Lipids (%)	6.50	7.21
	Others (%)	8.98	4.51
Output	Methanol utilization rate (mmol/gDCW/h)	1.24	1.45
	Carbon loss (mmolCO_2_/mmol methanol)	1.16	1.06

Based on these findings regarding cell wall composition, we further investigated the relationship between cell wall composition and environmental tolerance by comparing the growth of the Δ*PAS_0305* strain and the parental strain *P. pastoris* X33 on plates containing cell wall-destabilizing reagents Congo red (CR) and sodium dodecyl sulfate (SDS). As shown in Fig. 4c, d, the Δ*PAS_0305* strain exhibited faster growth on YPD + CR and YPD + SDS plates compared to *P. pastoris* X33, indicating that the Δ*PAS_0305* strain had greater resistance to cell wall-destabilizing reagents. Thus, disruption of the *PAS_0305* gene enhances the tolerance of *P. pastoris* cell walls to environmental stress.

The removal of the *PAS_0305* gene activated the cell wall sensors of *P. pastoris*, activating adaptations to environmental changes. Remodeling of the cell wall composition and the accumulation of trehalose are both responses to multiple environmental stresses after cell wall sensor activation. This is similar to the evolutionary mechanism of adaptive laboratory evolution, where effective modifications to the cell wall can artificially create new environmental pressures as continuous signals.

### Utilizing the superior phenotype of ***P. pastoris*** Δ***PAS_0305*** for SCP overproduction

According to the literature, methanol toxicity is a major challenge for improving the efficiency of methanol fermentation in *P. pastoris* [14]. Increased methanol tolerance has been shown to be crucial for enhancing methanol utilization in methylotrophs. Due to the enhanced robustness of strain Δ*PAS_0305* after activating cell wall sensors, it exhibited significantly faster growth compared to *P. pastoris* X33 at various methanol concentrations (1.5%, 2%, 2.5%, and 3%). Notably, the strain Δ*PAS_0305* achieved up to twofold higher OD_600_ values at a methanol concentration of 3.5% (Fig. 4b). These results demonstrate that both methanol tolerance and utilization in *P. pastoris* were enhanced after the *PAS_0305* gene was knocked out. To confirm that the growth difference was indeed due to the deletion of the *PAS_0305* gene, we constructed a *PAS_0305*-complementation strain, Δ*PAS_0305*-cp. The growth of the complemented strain was comparable to that of *P. pastoris* X33 at different methanol concentrations. At a methanol concentration of 1.5%, Δ*PAS_0305* exhibited a higher methanol utilization rate of 0.482 mM/gDW/h, a significant improvement over *P. pastoris* X33, which had a rate of 0.333 mM/gDW/h. These findings indicate that the absence of the *PAS-0305* gene promoted the growth of *P. pastoris* X33 on methanol. A previous study reported that the deletion of a glycosylphosphatidylinositol (GPI)-anchored protein increased the growth of *P. pastoris* GS115 when methanol was the sole carbon source [22], but there was no progress in understanding the underlying mechanisms involved in methanol utilization.

**Fig. 5:**
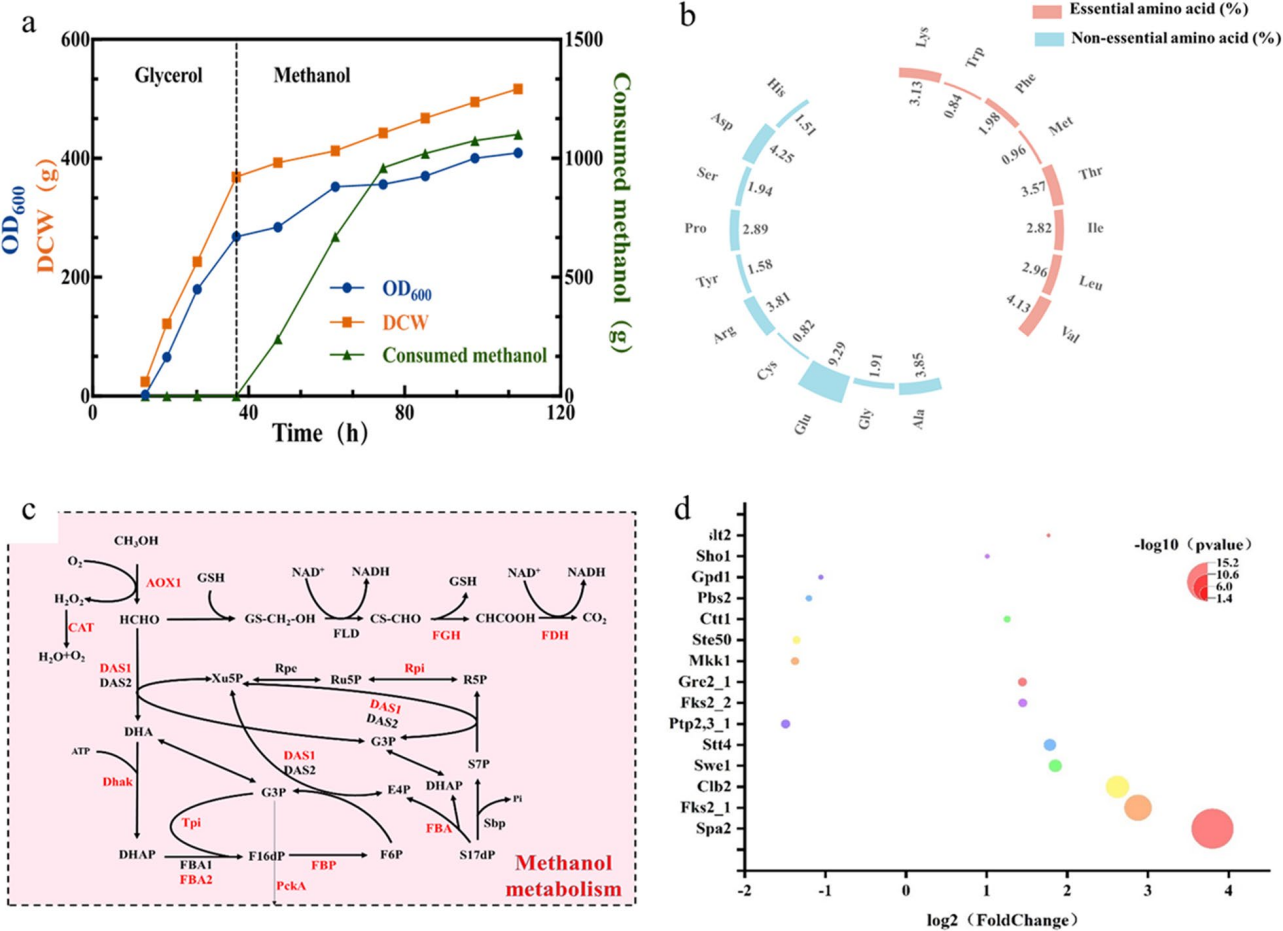
Analysis of fed-batch fermentation, nutrient consumption and signal transduction pathways of Δ*PAS_0305.* **a** Fed-batch fermentation of strain Δ*PAS_0305* in a 5-L bioreactor. **b** Amino acid content of Δ*PAS_0305* biomass*.* **c** Sketch of DEGs related to methanol metabolism. Genes in red showed significant upregulation in expression. **d** Comparison of the transcription levels of genes related to the MAPK signaling pathways

The utilization of methanol as a feedstock for the production of SCP in *P. pastoris* has been extensively studied [5]. One of the key nutritional aspects of SCP, either for human food or animal feed, is its high protein content. Here, high-density fermentation was employed to comprehensively examine the production process and evaluate the resulting SCP produced by *P. pastoris*. Compared with *P. pastoris* X33, the dry cell weight and crude protein content of the Δ*PAS_0305* strain respectively increased by 18.59% (from 102.2 g to 121.2 g/L) and 18.70% (from 56.62 to 67.21%) (Table 2). In the fermentation process, strain Δ*PAS_0305* achieved the highest methanol conversion ratio of 0.46 g DCW/g (Fig. 5a), which corresponds to 92% of the maximum theoretical yield (0.5 g DCW/g). This represents a significant improvement compared to the 0.37 g DCW/g achieved by *P. pastoris* X33. The absence of the gene *PAS_0305* in strain Δ*PAS_0305* led to a notable increase in the methanol utilization rate, resulting in enhanced dry cell weight and crude protein content. Conversely, the growth of the PAS-0305-complemented strain, Δ*PAS_0305*-cp, was similar to that of *P. pastoris* X33, resulting in comparable dry cell weight, crude protein content, and methanol conversion ratio. The Δ*PAS_0305* strain exhibited a 15.78% higher methanol utilization rate than *P. pastoris* X33. These findings were in line with the results of shake-flask fermentation. The disruption of the *PAS_0305* gene clearly contributed to the enhanced growth rate and biomass accumulation of *P. pastoris* when utilizing methanol. Increased crude protein content leads to improved nutritional value and quality of SCP in *P. pastoris*. Other yeast-based SCP sources such as *Saccharomyces cerevisiae*, *Yarrowia lipolytica*, and *Candida tropicalis* typically have crude protein contents ranging from 53 to 56% [23, 24], whereas *Haematococcus pluvialis* has a crude protein content of 64.9% [25]. However, SCP derived from Δ*PAS_0305* is a high-quality alternative protein rich in 8 essential amino acids of 20.39%, specially methionine (0.82%), threonine (2.06%) and lysine (2.83%) (Fig. 5b), whereby the latter is deficient in cereals. Thus, *P. pastoris* demonstrated significantly higher SCP production efficiency compared to traditional protein sources. This was primarily attributed to several key factors, including a shorter production time, greatly reduced land use, and the independence from weather conditions.

**Table 2:** Comparison of dry cell weight and crude protein of the *P. pastoris* ΔPAS_0305 knockout strain, the corresponding complementation (cp) strain, and the parental wild-type strain

Strains	Dry cell weight (g/L)	Crude protein (%)
*P. pastoris* X33	102.2	56.62
Δ*PAS_0305*	121.2	67.21
Δ*PAS_0305*-cp	102.1	55.98

### Comparative transcriptomic analysis and validation using genome-scale metabolic models (GSMM)

To further understand the mechanisms underlying the improved performance, we performed a comprehensive whole-transcriptome analysis to identify differentially expressed genes (DEGs) between the *P. pastoris* X33 strain and the Δ*PAS_0305* strain when cultivated in YPD medium. The strains exhibited significant differences in their transcriptomes, especially in pathways related to carbohydrate metabolism. Genes involved in methanol utilization, including both assimilation and dissimilation, were significantly upregulated in response to the rapid growth on methanol after knocking out the *PAS_0305* gene. Thus, the results confirmed that upregulation of methanol metabolism played an important role in enhancing the cell growth of Δ*PAS_0305* on methanol.

GSMMs can be used to predict the phenotype of a microorganism under a range of conditions, including those derived from genetic modification. A new version of GSMM (v3.0) for the growth of *P. pastoris* on glycerol and methanol was validated within the growth rate range. Based on the cell wall composition*,* GSMM could predict the metabolic phenotype of *P. pastoris*. The growth rates of *P. pastoris* X33 and the Δ*PAS_0305* strain were calculated using the model iMT1026 v3.0 [26]. Firstly, the biomass composition in iMT1026 v3.0 was adjusted according to the experimentally determined ratios (Table 1). According to these calculations, Flux Balance Analysis (FBA) was performed with the methanol utilization rate set at the experimentally measured value [27], i.e., 1.238 mmol methanol/gDCW/h for *P. pastoris* X33 and 1.454 mmol methanol/gDCW/h for Δ*PAS_0305*. The calculated growth rates were 0.0315 h^−1^ for *P. pastoris* X33 and 0.0335 h^−1^ for the Δ*PAS_0305* strain. Notably, these calculated results confirmed that deletion of *PAS_0305* could enhance the growth rate on methanol, which was related to specific metabolic adaptations of the Δ*PAS_0305* strain. The increase of the methanol utilization rate of Δ*PAS_0305* was in agreement with experimental data.

As metabolic flux distributions are sensitive to changes of biomass composition [28], we adapted the biomass equation to the modified composition of *P. pastoris*Δ*PAS_0305*, after which the GSMM could calculate the in vivo flux distribution accurately. The calculated flux distribution indicated that the carbon loss was 1.156 mmol CO_2_/mmol methanol for *P. pastoris* X33, compared to 1.056 mmol CO_2_/mmol methanol for Δ*PAS_0305* (Table 1). Based on these GSMM calculation results, the absence of Δ*PAS_0305* could reduce fluxes towards the dissimilatory pathway, leading to less carbon loss and higher methanol utilization efficiency compared with *P. pastoris* X33. These results confirmed that the internal metabolism of *P. pastoris* underwent significant changes, maximizing the flux toward biomass accumulation while minimizing the metabolic burden caused by the stress response to methanol.

### Underlying mechanism of cell wall sensor activation

The cell wall is the first line of defense of yeast cells against external stresses [16]. The yeast cell wall is a highly dynamic structure whose properties change constantly in response to stress, including adjustment of cell wall composition, or incorporation of new synthesized polysaccharides into the preexisting cell wall core. Therefore, the changes of cell wall permeability were perceived as a new environmental stress by *P. pastoris*, triggering cell wall sensor activation after knockout of Δ*PAS_0305.* MAPK signaling pathways play a crucial role in transmitting signals from the cell wall to the nucleus, ultimately regulating gene expression and cellular responses. These pathways are highly conserved across yeast species, making them a promising area of exploration for understanding cellular adaptation and stress responses [29]*.* Accordingly, it is crucial to investigate whether the signal transduction pathway has been affected in the Δ*PAS_0305* strain, as this could potentially explain the underlying mechanism, providing a new rational strategy for generating desirable phenotypes in *P. pastoris*.

**Fig. 6:**
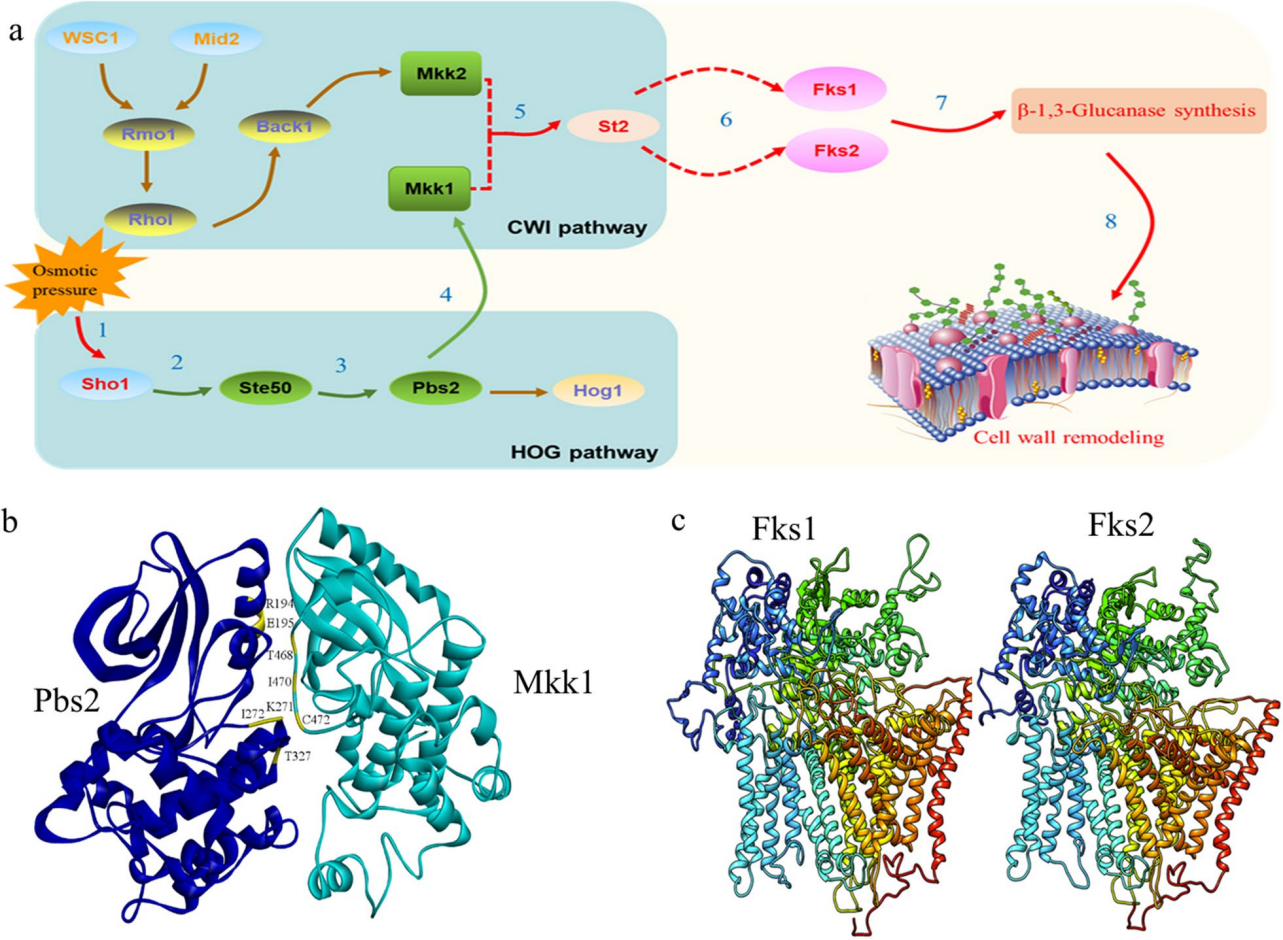
Analysis of signal transduction pathways of Δ*PAS_0305.* **a** Sketch of DEGs related to the CWI and HOG MAPK signaling pathways. Genes in green showed significant downregulation, while those in red showed significant upregulation. **b** Amino acid residues engaged in binding between Pbs2 and Mkk1. **c** Three-dimensional structure of FKS1 and FKS2 from *P. pastoris* modeled by SWISS-MODEL using *Saccharomyces cerevisiae* FKS1 as the template

As MAPK signaling pathways are highly conserved among yeast species, the key genes involved in the cell wall integrity (CWI) and high-osmolarity glycerol (HOG) pathways have been identified and annotated through comparative transcriptomics between *P. pastoris* X33 and Δ*PAS_0305* comparing whole-transcriptome comparison of *P. pastoris* X33 and Δ*PAS_0305* revealed that critical regulatory elements within CWI and HOG pathways exhibit variable transcriptional abundance (Fig. 5c, d). Firstly, the upregulation of Sho1 in response to hyperosmotic stress conditions in Δ*PAS_0305* was consistent with the observed increase of intracellular osmolytes due to enhanced cell wall permeability. This suggests that Sho1 plays a role as a sensor in the stress response of Δ*PAS_0305*. Secondly, the Rho GTPase Ste50 was downregulated in the sho1 branch, which synchronously led to the downregulation of pbs2 transcription. This result suggested that the Hog signaling pathway was perturbed. However, there is a lack of detailed understanding regarding the connection between the HOG and CWI pathways [30]. Previous studies demonstrated that the MAP kinases Mkk1 and Pbs2 of the HOG pathway, as well as Slt2 and Hog1 of the CWI interact physically, forming a complex [31]. In Δ*PAS_0305*, a positive correlation between Pbs2 and Mkk1 transcription was observed (Fig. 6), indicating similar perturbations in response to cell wall sensor activation. These findings shed new light on the interplay between the HOG and CWI pathways, suggesting that alterations in one pathway can influence the other. However, further studies are needed to unravel the precise mechanisms underlying these connections, as well as their implications for cell wall dynamics and the stress response of *P. pastoris*.

To investigate the potential interaction between Pbs2 and Mkk1, we utilized RosettaDock, a protein docking and simulation tool [32]. According to the docking results, the* P *value between Pbs2 and Mkk1 was less than 0.5, indicating that the results are credible. The ΔG of the composite system was − 9.7, which indicated that the binding between Pbs2 and Mkk1 is spontaneous. The protein docking complex revealed that approximately 15.4% of amino acid residues in Pbs2 and 13.6% of those in Mkk1 were involved in the binding interface. Additionally, the binding area of Pbs2 was found to be marginally larger than that of Mkk1, with the latter occupying 9.9% and Pbs2 occupying 10.7% of the binding interface (Fig. 6b). Notably, the interaction between Pbs2 and Mkk1 was stabilized by four specific amino acids (Mkk1: F198, R275; Pbs2: R323, G471), which formed stable hydrogen bonds (Fig. 6b). This indicates a strong interaction between Pbs2 and Mkk1. The consistency between the transcriptional changes and the molecular docking results further supports the hypothesis of a signaling interaction between Pbs2 and Mkk1, bridging the HOG and CWI pathways. These findings also provide valuable insights into the molecular mechanisms underlying the cross-talk between the HOG and CWI pathways in response to hyperosmotic stress.

The Pbs2-Mkk1 signaling pathway was found to transmit the signal from the HOG to the CWI MAPK module, confirming that activation of the CWI pathway depended on signal transduction via the HOG pathway. Finally, the downregulation of MAPKs Mkk1/Mkk2 led to the activation of the MAPK Slt2, thereby activating the CWI signaling pathway. In the Δ*PAS_0305* strain, the transcription of Slt2 was indeed upregulated, while other phosphatases such as Ptp2/Ptp3 were downregulated. This result was in agreement with previous reports that Slt2 activity can be negatively regulated by dephosphorylation through the activity of different phosphatases such as Ptp2 and Ptp3 [33, 34]. It is possible that Slt2 is activated by either the CWI signal or the pbs2-Mkk1 mediated HOG signal. The knockout of the *PAS_0305* gene in *P. pastoris* resulted in sequential signal transduction through two MAPK pathways, leading to the elicitation of a rescue response, including the remodeling of the cell wall.

Significantly, whole-transcriptome analysis showed that FKS1 and FKS2 were respectively upregulated by 2.8- and 1.7-fold. The FKS family genes are responsible for β-1,3-glucan synthesis [35]. The amino acid sequences of FKS1 and FKS2 from *P. pastoris* X33 showed a sequence identity of 74.95% and 54.99% respectively, when compared to FKS1 from *S. cerevisiae* (Fig. 6c). A high-resolution cryo-electron microscopy structure of *S. cerevisiae* FKS1 was recently reported, and it was confirmed to participate in β-1,3-glucan synthesis as well as affect the lipid composition, resulting in drug-resistance of fungi [36]. Here, we also obtained similar results, whereby the upregulation of FKS in the Δ*PAS_0305* strain elicited cell wall remodeling by increasing the β-1,3-glucan and lipid content, thus improving yeast robustness. Notably, stress resistance may be the result of fungal responses to external signals, transduced either through the CWI pathway or the pbs2-Mkk1 mediated HOG pathway. In a previous study, activation of the CWI pathway by different transcriptional factors was confirmed to improve the robustness of *S. cerevisiae* [36]. This mechanism could also explain why the Δ*PAS_0305* strain exhibited a remodeled cell wall composition and improved robustness after activating cell wall sensors.

## Discussion

Microorganism-derived SCPs can be produced in abundance and contain essential amino acids, making them a promising alternative protein source that does not affect the supply of food sources such as soy, meat, milk, and fish [37]. SCP produced from methanol using *P. pastoris* offers an attractive alternative to animal-derived protein due to the rapid production rate, lower space requirements, independence of climate or seasons and more sustainable production process [37]. Moreover, methanol can be industrially produced from CO_2_ by photocatalytic or electrochemical reduction, which may contribute to SCP overproduction with a (nearly) zero CO_2_ footprint. Stoichiometric models of *P. pastoris* showed that 18% of the used methanol was dissimilated by yeast cells [38], while the value could reach 70–80% in fed-batch fermentations [6]. However, excessive dissimilation of methanol greatly increases carbon losses. The complexity of the metabolic network of *P. pastoris* made it difficult to transform a single metabolic pathway without affecting another. It has been reported that simply knocking out the dissimilation pathway may not be the best solution, because this would lead to a significant loss of cell robustness [39]. Therefore, the construction of SCP overproduction strains based on *P. pastoris* is time-consuming and laborious since it requires multiple rounds of genetic manipulation, which face the challenge of balancing methanol metabolism and biomass biosynthesis.

In this study, cell wall sensor activation was used as an effective breeding strategy for improving the methylotrophic performance of *P. pastoris.* The global metabolism of *P. pastoris* was successfully reprogrammed for SCP overproduction, which provided a new theoretical basis for understanding the mechanisms of methanol utilization and carbon loss in *P. pastoris* via unlocking cell wall sensors. This research provides new insights into cellular mechanisms underlying the response to environmental stress, which resulted in a superior methylotrophic fermentation performance of *P. pastoris*. Thus, the novel strategy can provide a new perspective for the construction of versatile *P. pastoris* cell factories to produce a variety of chemicals based on the flux balance response to cell wall stress.

Currently, the production of well-balanced animal feed heavily relies on corn kernel and soybean proteins, with almost all imported soybean in China being used as a protein source for animal feed. However, this reliance on plant-based proteins poses challenges due to the low energy efficiency of plant photosynthesis, as well as significant freshwater consumption and increasing land use. To address these challenges, the internal metabolism of *P. pastoris* has been thoroughly reprogrammed to maximize the flux towards biomass accumulation. This optimization resulted in impressive crude protein and cell dry weight yields of 67.21% and 121.2 g/L, respectively. The single-cell protein (SCP) produced by the Δ*PAS_0305* strain is competitive with soy (38.6%), fish (17.8%), meat (21.2%), and whole milk (3.28%) [40]. Notably, the methanol conversion rate of the engineered *P. pastoris* reached the state-of-the-art level of 0.46 g/g, amounting to 92% of the maximum theoretical yield. This biotechnological strategy resulted in a significant advancement in the directed conversion of methanol into biomass. The impressive advancements in the space–time productivity of SCP in *P. pastoris* have positioned it as a cost-effective option for the industrial production of protein.

Further analysis of the strain has shed new light on the mechanisms involved in unlocking cell wall sensors to maximize the flux from methanol towards biomass accumulation. This novel strategy has revealed the potential of manipulating cellular signaling pathways to optimize metabolic networks and achieve exceptional phenotypic characteristics, thereby providing new strategies for constructing versatile cell factories in *P. pastoris*. By addressing the issues of methanol fermentation efficiency and carbon loss in the directed conversion of methanol into SCP, this approach represents a great advance in cost-competitive industrial bio-manufacturing of microbial protein, thereby contributing to global food security.

## Materials and methods

### Strains and culture conditions

All strains and plasmids used in this study are listed in Additional file 2: Extended Data Table S2. Unless otherwise specified, *P. pastoris* strains were cultivated in YPD medium, which was supplemented with 100 mg/L zeocin to screen transformants. Delft basic salt medium containing 7.5 g/L (NH_4_)_2_SO_4_, 14.4 g/L KH_2_PO_4_, 0.5 g/L MgSO_4_•7H_2_O, 1 mL/L vitamin solution, and 2 mL/L PTM1 trace salts solution was used for cell cultivation with methanol as sole carbon sources [41]. *E. coli* DH5α used as the host for plasmid construction and was cultured at 37 °C in LB medium.

### Transmission electron microscopy

To assess the thickness of the cell wall by transmission electron microscopy, *P. pastoris* cells were grown at 30 °C and 220 rpm to the exponential phase in Delft basic salt medium containing 0.5% or 3% methanol. The cells were harvested by centrifugation at 5000×*g* for 10 min, washed three times, and resuspended in phosphate buffer (pH = 7.4, 50 mM). The suspension was premixed with an equal volume of 2.5% glutaraldehyde for 12 h at 4 °C and subsequently dehydrated with a graded series of 25, 50, 70, 80, 95, and 100% ethanol solution. Pure ethanol was then changed to propylene oxide and specimens were gradually infiltrated with increasing concentrations (30%, 50%, 70% and 100%) of Agar 100 epoxy resin mixed with propylene oxide for a minimum of 3 h per step. Samples were embedded in pure, fresh Agar 100 epoxy resin and polymerized at 60 °C for 48 h. Ultrathin sections were stained for 3 min with lead citrate and viewed with an JEM-1230 electron microscope.

### Illumina sequencing and analysis of significantly differentially expressed genes

The total RNA was extracted from the cells grown to the exponential phase under the two indicated culture conditions using TRIzol reagent (Invitrogen, Carlsbad, CA, USA), after which the two sequencing libraries were constructed using the TruSeq™ Stranded Total RNA Library Prep Kit for Illumina (Illumina, San Diego, CA, USA) following the manufacturer’s protocol. After cluster generation, the library preparations were sequenced using the Illumina HiSeq 2000 platform, which generated 150 bp paired-end reads. The significantly differentially expressed genes were identified using the inclusion criteria |log_2_FC| ≥ 1 and *P* < 0.05.

### Construction of strains and plasmids

The method used for chromosomal gene deletion in *P. pastoris* was based on the CRISPR–Cas9 meditated genome editing system as described previously [11]. The plasmid pPICZ-Cas9-gGUT1, which expresses Cas9 and GUT1-gRNA under the control of the bidirectional promoter P_HTX1_ [11], was used as template to construct the genome editing vector pPICZCas9-g (target genes) by Gibson assembly. A *PAS_0305*-complementation strain, Δ*PAS_0305*-cp, was generated by reintegrating the knocked-out Δ*PAS_0305* gene into its original position in the genome of strains Δ*PAS_0305*. The mutants were screened by colony PCR and further confirmed by gene sequencing. All primers used for strain and plasmid construction are listed in Additional file 3: Extended Data Table S3.

To detect the secretion of a heterologously expressed protein for verifying cell wall permeability, the gene encoding endoglucanase HgCel5A from *Humicola grisea* (Accession number: KX096883.1) was synthesized by Genewiz Biotech Co., Ltd. (Suzhou, China), and cloned into the expression vector with P_AOX_ promoter and T_GAP_ terminator.

### Transformation and selection of *P. pastoris* clones

The fragment was linearized by *Avr*II restriction digestion and then electroporated into *P. pastoris* X33 using a MicroPulser electroporator (BIO-RAD). Positive clones were selected on YPD plates with 100 mg/L zeocin for 48–96 h at 30 °C. The recombinant clones were screened by colony PCR and confirmed by DNA sequencing, followed by sub-culture on YPD plates with increasing zeocin concentrations (100, 200, 300, 400, and 500 mg/L) for 48 h at 30 °C each. The colonies that grew well under high selection pressure were selected for shake-flask fermentation.

### Fed-batch fermentation

After overnight cultivation in YPD medium at 30 °C, the seed cultures were inoculated (8%, v/v) into a 5-L bioreactor containing 3 L of basal salt medium (BSM) supplemented with 4.35 mL/L of PTM1 trace salts. The fermentation process consisted of three stages. In Stage I, the focus was on cell growth, and the dissolved oxygen (DO) level was maintained above 30% of air saturation by adjusting the agitation rate (200–800 rpm). In Stage II, a 50% (w/w) glycerol solution (containing 12 mL/L of PTM1 solution) was continuously fed to the bioreactor to maintain the dissolved oxygen level above 30%. The temperature was controlled at 30 °C using recirculating water, and the pH was maintained at 5.5 by adding 25% ammonium hydroxide during stages I and II.

In Stage III, protein production was induced by adding methanol (containing 12 mL/L of PTM1 solution). The culture temperature was lowered to 28 °C, the pH was adjusted to 6.0, and the DO level was maintained above 20% of air saturation with an agitation rate of 800 rpm.

### Biomass and crude protein analysis

The biomass was characterized by measuring the dry cell weight (DCW, gL^−1^) and absorbance at 600 nm [42]. Cell pellets from 3 mL culture were washed with sterile water three times and resuspended in 1 mL of sterile water. The tubes containing centrifuged cells were dried at 105 °C until a constant weight was maintained. The nitrogen content of the homogenized residues was analyzed using the Kjeldahl method [43]. A conversion factor of 6.25 was used to calculate the theoretical protein content from the nitrogen content.

### Enzyme activity assays

CMCase activity was measured colorimetrically, using a 3,5-dinitrosalicylic acid (DNS) assay with carboxymethyl cellulose (CMC) as the substrate [44].

### Sodium dodecyl sulfate polyacrylamide gel electrophoresis (SDS-PAGE)

The extracellular proteins were analyzed using SDS-PAGE. The purified proteins were separated using 12% separating gel with 0.1% SDS and 5% stacking gel. The protein bands were stained with Coomassie brilliant blue R-250.

### Analysis of cell wall components in recombinant *P. pastoris*

The total cellular β-1,3 glucan and chitin contents of *P. pastoris* cells were quantitatively analyzed according to the NREL Laboratory Analytical Procedures for biomass using a two-step acid method. Lipids were extracted and quantified by (UPLC)-Q Exactive HF MS (Thermo Fisher Scientific).

### Disaccharide identification and trehalose measurement

The contents of osmolytes was determined using high-performance liquid chromatography (Shimadzu, Kyoto, Japan) with a refractive index detector (Shimadzu) on an Aminex HPX-42A column (Bio-Rad, Hercules, CA, USA) as the mobile phase at a flow rate of 0.5 mL/min at 35 °C. The disaccharides were analyzed using an ultra-performance liquid chromatography (UPLC) system (Nexera 30A, Shimadzu, Kyoto, Japan) coupled with a mass spectrometer (TripleTOF™ 5600, Applied Biosystem Sciex, United States) in negative electrospray ionization (ESI) mode. The disaccharide was identified using an LC equipped with a SeQuant ZIC-HILIC column (100 × 2.1 mm, 3.5 μm, Merck, Germany) [45].

The intracellular trehalose content was measured using the modified anthrone method as described previously [46]. Aliquots of 200 µL were mixed with 1 mL of cold anthrone reagent and incubated at 100 °C for 10 min prior to the measurement of A_620_.

### Propidium iodide and Nile red staining

To verify cell wall permeability, *P. pastoris* cells were grown in YPD to an OD_600_ of approximately 1.0, washed twice with phosphate-buffered saline (PBS, pH 7.4) and resuspended in the same buffer [47]. After 5 min at room temperature, 200 μL of the cell suspension were added in triplicate to a 96-well fluorescence plate, followed by the addition of propidium iodide or Nile red stock solution (0.5 mg/mL) prepared in advance according to the calculated volume (5 μL). Then, the initial fluorescence was measured and the black fluorescence plate placed on a shaker at 150 rpm and 30 °C. Propidium iodide fluorescence was measured at an excitation wavelength of 535 nm and an emission wavelength of 615 nm, while Nile red was measured at an excitation wavelength of 540 nm and an emission wavelength of 630 nm.

### Methanol tolerance assay

*Pichia pastoris* cells were grown in liquid YPD to an OD_600_ of approximately 0.4, after which equal volumes of the culture (10 μL) were transferred to a 96-well fluorescence plate containing YPD medium with 1.5%, 2%, 2.5%, and 3.5% methanol, respectively. The fluorescence plate was then incubated in a 30 °C shaker at 150 rpm. Starting at 24 h, the OD_600_ was measured every 1 h to plot growth curves [48].

### SDS and CR sensitivity assay

The strains were grown in liquid YPD at 30 °C overnight. Then, 6.1 × 10^7^ cells from each strain were subjected to tenfold serial dilution in sterile water. Aliquots (1 μL) from each dilution were then spotted onto YPD agar plates containing 50 μg/mL Congo red (CR) or 50 μg/mL sodium dodecyl sulfate (SDS) and cultured at 30 °C for 48 h [49].

### Numerical analysis of *P. pastoris* growth

The optimal growth rate and flux distribution were calculated based on the biomass objective function using the genome-scale metabolic model iMT1026 v3.0 of *P. pastoris* by conducting flux balance analysis (FBA). The COBRApy toolbox was used to perform FBA in Python [50]. CPLEX (IBM, Armonk, NY, USA) was used as the linear programming solver. For the simulation, glycerol was used as supplementary carbon source, and the uptake rate was set at 0.502 mmol gDCW^−1^ h^−1^. The sources of nitrogen, oxygen, phosphorus and sulfur were not constrained.

### Protein docking

The homology modeling of Mkk1 and Pbs2 was performed using SWISS-MODEL (https://swissmodel.expasy.org/). A rigid docking method was used to determine the spatial position and pose of the protein molecules. Both PDB structures for the proteins were submitted to the ClusPro online server (https://cluspro.bu.edu/), and the highest-scoring initial docking result was chosen [51]. Fine docking was performed using RosettaDock for molecular contact localization.

### Supplementary Information

**Additional file 1**. Identification of significantly differentially expressed genes by RNA-seq in response to changes of methanol stress. 

**Additional file 2**. Strains and plasmids used in this study.

**Additional file 3**. Primers used for strain and plasmid construction in this study. 

## Data Availability

All data generated or analyzed during this study are included in this article.
