# Supplementary material for: Deciphering cell wall sensors enabling the construction of robust P. pastoris for single-cell protein production
Source: Biotechnol Biofuels Bioprod. 2023 Nov 17;16:178. doi: 10.1186/s13068-023-02428-7 (PMC10655344; doi:10.1186/s13068-023-02428-7)
Supplement: Supplementary file 1 — Additional file 1. Identification of significantly differentially expressed genes by RNA-seq in response to changes of methanol stress. [file 13068_2023_2428_MOESM1_ESM.docx]

**Table S1 Identification of significantly differentially expressed genes by RNA-seq**

| Gene name | Gene description | 0.5% methanol-mean TPM | 3% methanol-mean TPM | FC (3% methanol /0.5% methanol) | Log_2_FC (3% methanol /0.5% methanol) | *P* value |
| --- | --- | --- | --- | --- | --- | --- |
| PAS_chr3_0030 | Hypothetical protein | 168.4633 | 1682.98 | 8.86 | 3.147341 | 1.72E-127 |
| PAS_chr4_0151 | Hypothetical protein | 627.3067 | 118.72 | 0.167 | -2.58393 | 7.09E-118 |
| PAS_chr4_0152 | Hypothetical protein | 1363.34 | 258.4233 | 0.166 | -2.58878 | 1.83E-112 |
| PAS_chr1-4_0226 | hypothetical protein | 13.07667 | 161.1033 | 11.073 | 3.469025 | 2.44E-108 |
| PAS_chr3_0015 | Hypothetical protein | 47.35 | 341.03 | 6.316 | 2.6589 | 4.47E-105 |
| PAS_chr4_0147 | Aromatic aminotransferase II | 13.60667 | 152.34 | 9.949 | 3.314563 | 1.06E-103 |
| PAS_FragB_0003 | Rho GTPase activating protein | 12.38667 | 81.9 | 5.83 | 2.543503 | 7.80E-90 |
| PAS_chr4_0627 | Plasma membrane localized protein that protects membranes from desiccation | 2636.09 | 14490.7 | 5.048 | 2.335846 | 1.67E-87 |
| PAS_chr4_0360 | Hypothetical protein | 947.1667 | 148.02 | 0.139 | -2.84643 | 5.60E-81 |
| PAS_chr4_0363 | Hypothetical protein | 77.9 | 15.97 | 0.18 | -2.47238 | 1.69E-70 |
| PAS_chr1-3_0169 | Hypothetical protein | 24.22 | 151.2567 | 5.596 | 2.484436 | 2.17E-62 |
| PAS_chr2-2_0113 | Inositol 1-phosphate synthase | 4.133333 | 37.91333 | 8.028 | 3.005124 | 4.02E-61 |
| PAS_chr2-2_0267 | 3-ketoacyl-CoA thiolase with broad chain length specificity | 51.54667 | 214.61 | 3.688 | 1.882684 | 8.92E-61 |
| PAS_chr3_0987 | Mitochondrial aldehyde dehydrogenase | 6.783333 | 42.41667 | 5.55 | 2.472489 | 2.92E-59 |
| PAS_chr4_0828 | Myo-inositol transporter with strong similarity to the minor myo-inositol transporter Itr2p | 170.57 | 671.5433 | 3.465 | 1.792778 | 7.16E-59 |
| PAS_chr1-4_0584 | Lectin-like protein with similarity to Flo1p, thought to be expressed and involved in flocculation | 102.8 | 513.35 | 4.445 | 2.152096 | 2.22E-55 |
| PAS_chr1-4_0691 | hypothetical protein | 71.64 | 297.95 | 3.678 | 1.879101 | 5.96E-49 |
| PAS_chr3_0016 | Hypothetical protein | 20.71333 | 90.18 | 3.842 | 1.941801 | 8.29E-44 |
| PAS_chr4_0540 | Zinc cluster transcriptional activator | 18.30667 | 4.356667 | 0.208 | -2.26192 | 1.16E-41 |
| PAS_chr3_0299 | Aspartic protease, attached to the plasma membrane via a glycosylphosphatidylinositol (GPI) anchor | 64.94333 | 18.92333 | 0.255 | -1.97127 | 2.38E-39 |
| PAS_chr3_0440 | Lactate transporter | 122.2333 | 379.5567 | 2.742 | 1.455436 | 3.07E-39 |
| PAS_chr4_0314 | Phenylpyruvate decarboxylase, catalyzes decarboxylation of phenylpyruvate to phenylacetaldehyde | 6.44 | 31.04 | 4.201 | 2.07061 | 5.06E-39 |
| PAS_chr4_0851 | Hypothetical protein | 409.62 | 147.0233 | 0.32 | -1.64266 | 3.03E-38 |
| PAS_chr4_0375 | Hypothetical protein | 188.8067 | 50.99333 | 0.241 | -2.05473 | 3.77E-38 |
| PAS_chr1-4_0410 | Putative positive regulator of mannosylphosphate transferase (Mnn6p) | 3.04 | 15.35 | 4.42 | 2.144183 | 1.57E-31 |
| PAS_chr4_0439 | Plasma membrane transporter for both urea and polyamines | 9.72 | 35.91667 | 3.26 | 1.704965 | 3.53E-28 |
| PAS_chr3_1157 | hypothetical protein | 170.1767 | 74.76333 | 0.386 | -1.37259 | 1.04E-27 |
| PAS_chr1-4_0582 | Hypothetical protein | 3.19 | 14.99333 | 4.164 | 2.057966 | 2.99E-27 |
| PAS_chr1-1_0030 | General amino acid permease | 58.50333 | 175.8433 | 2.666 | 1.414412 | 1.88E-26 |
| PAS_chr3_0075 | Protein of unknown function | 21.33333 | 60.46 | 2.5 | 1.321976 | 6.64E-26 |
| PAS_chr3_0134 | Hypothetical protein | 18.55333 | 7.403333 | 0.351 | -1.50951 | 1.51E-25 |
| PAS_chr2-1_0887 | hypothetical protein | 671.67 | 1764.79 | 2.333 | 1.22197 | 2.37E-25 |
| PAS_chr4_0305 | O-glycosylated protein required for cell wall stability | 55.68667 | 175.5933 | 2.814 | 1.492579 | 6.14E-25 |
| PAS_chr4_0043 | Mitochondrial aldehyde dehydrogenase | 538.83 | 1719.583 | 2.841 | 1.506453 | 1.07E-24 |
| PAS_chr1-1_0173 | Hypothetical protein | 29.76 | 124.7 | 3.868 | 1.951676 | 9.51E-23 |
| PAS_chr2-2_0208 | Hypothetical protein | 184.16 | 658.4167 | 3.223 | 1.688555 | 1.60E-22 |
| PAS_chr3_0877 | Protein with a potential role in cell survival pathways, required for the diauxic growth shift | 138.76 | 69.87667 | 0.444 | -1.17276 | 1.68E-22 |
| PAS_chr4_0914 | hypothetical protein | 75.42667 | 272.65 | 3.306 | 1.7253 | 5.46E-22 |
| PAS_chr3_0662 | Ferrioxamine B transporter | 53.93 | 180.69 | 2.994 | 1.582188 | 1.53E-21 |
| PAS_chr2-1_0016 | 2-deoxyglucose-6-phosphate phosphatase, similar to Dog2p, member of a family of low molecular weight | 125.71 | 58.40667 | 0.415 | -1.26878 | 1.63E-21 |
| PAS_chr1-4_0295 | Alpha subunit of fatty acid synthetase | 85.12 | 37.90333 | 0.387 | -1.37067 | 2.39E-21 |
| PAS_chr1-1_0357 | NADPH-dependent medium chain alcohol dehydrogenase with broad substrate specificity | 2.49 | 12.36333 | 4.422 | 2.14456 | 3.69E-21 |
| PAS_chr3_0482 | Putative alanine transaminase (glutamic pyruvic transaminase) | 476.6433 | 248.6767 | 0.462 | -1.11262 | 6.57E-20 |
| PAS_chr1-1_0486 | hypothetical protein | 189.82 | 91.41333 | 0.43 | -1.21768 | 8.70E-20 |
| PAS_chr3_0438 | Member of the PUF protein family, which is defined by the presence of Pumilio homology domains | 20.33 | 51.90667 | 2.241 | 1.164325 | 1.39E-19 |
| PAS_chr2-2_0199 | One of two identical histone H3 proteins (see also HHT2) | 2674.853 | 1456.037 | 0.497 | -1.00814 | 1.49E-19 |
| PAS_chr1-4_0290 | Hypothetical protein | 19.32667 | 64.82333 | 2.975 | 1.572774 | 2.45E-19 |
| PAS_chr4_0624 | Non-essential protein of unknown function required for transcriptional induction | 1418.41 | 728.06 | 0.456 | -1.13336 | 2.64E-19 |
| PAS_chr2-1_0649 | Acetate transporter required for normal sporulation | 10.36667 | 27.15333 | 2.308 | 1.206802 | 6.21E-19 |
| PAS_chr3_0781 | Glycogen synthase, similar to Gsy1p | 360.1467 | 203.93 | 0.498 | -1.0045 | 7.54E-19 |
| PAS_chr4_0009 | Hypothetical protein | 61.12333 | 163.0967 | 2.377 | 1.249056 | 9.06E-19 |
| PAS_chr1-1_0107 | NADP(+)-dependent glutamate dehydrogenase | 1826.337 | 875.6233 | 0.427 | -1.22793 | 9.94E-19 |
| PAS_chr1-4_0517 | Subunit of both RNase MRP, which cleaves pre-rRNA, and nuclear RNase P | 13.61 | 37.21667 | 2.419 | 1.274355 | 4.63E-18 |
| PAS_chr4_0361 | Hypothetical protein | 628.5833 | 327.79 | 0.463 | -1.11005 | 5.01E-18 |
| PAS_chr2-2_0482 | hypothetical protein | 80.73333 | 0.966667 | 0.01 | -6.64035 | 5.18E-18 |
| PAS_chr2-1_0237 | Putative protein of unknown function | 72.57333 | 33.73667 | 0.411 | -1.28391 | 5.31E-18 |
| PAS_chr1-1_0008 | Beta subunit of fatty acid synthetase; catalyzes the synthesis of long-chain saturated fatty acids | 101.2333 | 47.20333 | 0.405 | -1.30544 | 7.51E-18 |
| PAS_chr3_1068 | Protein of unknown function that associates with ribosomes | 346.1867 | 922.6 | 2.423 | 1.276943 | 8.33E-18 |
| PAS_chr1-1_0482 | hypothetical protein | 75.39333 | 223.0833 | 2.668 | 1.415813 | 1.05E-17 |
| PAS_chr3_0188 | Major of three pyruvate decarboxylase isozymes | 1395.433 | 787.71 | 0.499 | -1.00179 | 1.26E-17 |
| PAS_chr2-1_0323 | Essential Hsp90p co-chaperone | 275.1367 | 140.35 | 0.46 | -1.11952 | 1.53E-17 |
| PAS_chr4_0860 | Hypothetical protein | 92.26333 | 228.51 | 2.232 | 1.158361 | 2.65E-17 |
| PAS_chr4_0399 | Ferric reductase and cupric reductase | 8.363333 | 22.08667 | 2.336 | 1.22406 | 3.26E-17 |
| PAS_chr2-2_0329 | Adenylosuccinate lyase, catalyzes two steps in the 'de novo' purine nucleotide biosynthetic pathway | 454.3533 | 251.87 | 0.491 | -1.02646 | 3.37E-17 |
| PAS_chr3_0778 | Hypothetical protein | 221.39 | 555.2667 | 2.217 | 1.14846 | 5.19E-17 |
| PAS_chr1-4_0538 | Fatty-acyl coenzyme A oxidase | 42.92 | 111.1067 | 2.28 | 1.188964 | 5.76E-17 |
| PAS_chr1-3_0206 | Permease of basic amino acids in the vacuolar membrane | 8.233333 | 22.14667 | 2.361 | 1.239406 | 5.92E-17 |
| PAS_chr3_0456 | Glycolytic enzyme phosphoglucose isomerase | 525.6067 | 291.8333 | 0.492 | -1.02402 | 1.14E-16 |
| PAS_chr3_1184 | hypothetical protein | 15.14333 | 39.18 | 2.29 | 1.195115 | 2.33E-16 |
| PAS_chr3_0418 | Zinc-finger inhibitor of HO transcription | 36.74333 | 89.30667 | 2.158 | 1.109891 | 3.89E-16 |
| PAS_chr4_0042 | Hypothetical protein | 35.98 | 115.4133 | 2.911 | 1.541376 | 4.25E-16 |
| PAS_chr1-3_0153 | Plasma membrane pyridoxine (vitamin B6) transporter | 8.976667 | 26.65333 | 2.641 | 1.401313 | 4.44E-16 |
| PAS_chr4_0148 | Gamma-tubulin | 3.763333 | 12.37 | 2.904 | 1.538125 | 7.43E-16 |
| PAS_chr2-2_0299 | Plasma membrane ATP binding cassette (ABC) transporter | 5.573333 | 15.13667 | 2.367 | 1.243286 | 1.09E-15 |
| PAS_chr2-1_0454 | Major exo-1,3-beta-glucanase of the cell wall, involved in cell wall beta-glucan assembly | 38.01667 | 85.97 | 2.003 | 1.002486 | 1.35E-15 |
| PAS_chr1-4_0681 | hypothetical protein | 5.97 | 19.46333 | 2.867 | 1.519354 | 1.72E-15 |
| PAS_chr4_0945 | hypothetical protein | 64.31 | 146.87 | 2.017 | 1.012042 | 2.78E-15 |
| PAS_chr1-1_0122 | Hypothetical protein | 106.5233 | 39.66667 | 0.329 | -1.60411 | 3.08E-15 |
| PAS_chr3_0287 | Integral membrane protein localized to mitochondria (untagged protein) and eisosomes, immobile patch | 1699.66 | 4473.06 | 2.36 | 1.238844 | 3.13E-15 |
| PAS_chr3_0024 | Plasma membrane transporter for both urea and polyamines, expression is highly sensitive to nitrogen | 12.4 | 32.12 | 2.309 | 1.206981 | 3.29E-15 |
| PAS_chr4_0003 | Hypothetical protein | 3.16 | 11.15 | 3.109 | 1.636396 | 3.29E-15 |
| PAS_chr3_0648 | Thiazole synthase, catalyzes formation of the thiazole moiety of thiamin pyrophosphate | 1506.143 | 3460.87 | 2.053 | 1.037483 | 5.20E-15 |
| PAS_chr2-1_0192 | Protein of unknown function | 84.65333 | 248.6767 | 2.575 | 1.364537 | 6.17E-15 |
| PAS_chr2-1_0368 | Isopropylmalate isomerase, catalyzes the second step in the leucine biosynthesis pathway | 89.31 | 47.77333 | 0.467 | -1.09895 | 1.33E-14 |
| PAS_chr1-4_0164 | Protein of unknown function, has similarity to Pry1p and Pry3p | 63.98667 | 146.4733 | 2.048 | 1.034302 | 2.59E-14 |
| PAS_chr1-1_0323 | Diadenosine 5',5''-P1,P4-tetraphosphate phosphorylase II (AP4A phosphorylase) | 140.68 | 76.41667 | 0.483 | -1.04916 | 9.31E-14 |
| PAS_chr4_0112 | Threonine aldolase | 354.0133 | 839.78 | 2.122 | 1.085374 | 9.44E-14 |
| PAS_chr2-1_0566 | Vacuolar protein of unknown function | 9.37 | 26.23333 | 2.463 | 1.300677 | 1.18E-13 |
| PAS_chr2-1_0229 | Protein binding phosphatidylinositol 3-phosphate, involved in telomere-proximal repression of gene e | 256.0633 | 135.27 | 0.468 | -1.09498 | 2.10E-13 |
| PAS_chr3_0854 | Hypothetical protein | 121.9933 | 50.59667 | 0.373 | -1.42224 | 3.28E-13 |
| PAS_chr2-1_0092 | Pho85 cyclin of the Pcl1,2-like subfamily, involved in entry into the mitotic cell cycle and regulat | 125.4167 | 324.68 | 2.282 | 1.190486 | 4.70E-13 |
| PAS_chr4_0688 | Mitochondrial succinate-fumarate transporter | 259.3367 | 143.09 | 0.492 | -1.02235 | 6.66E-13 |
| PAS_chr3_0260 | Hypothetical protein | 73.11 | 41.54667 | 0.499 | -1.00281 | 2.08E-12 |
| PAS_chr3_0006 | NADPH-dependent medium chain alcohol dehydrogenase | 108.58 | 256.9267 | 2.108 | 1.075541 | 2.15E-12 |
| PAS_chr4_0756 | Mitochondrial protein kinase | 9.49 | 4.443333 | 0.413 | -1.27637 | 2.28E-12 |
| PAS_chr3_1024 | NAD(+)-dependent glutamate synthase (GOGAT) | 68.04 | 38.66667 | 0.494 | -1.01602 | 5.02E-12 |
| PAS_chr3_0408 | Hypothetical protein | 1391.523 | 689.8067 | 0.442 | -1.17676 | 5.19E-12 |
| PAS_chr1-4_0330 | Hypothetical protein | 146.4233 | 384.3467 | 2.338 | 1.225015 | 5.66E-12 |
| PAS_chr3_0377 | Widely conserved NADPH oxidoreductase containing flavin mononucleotide (FMN) | 9.72 | 22.99667 | 2.091 | 1.06415 | 6.82E-12 |
| PAS_chr3_0970 | Hypothetical protein | 35.00333 | 94.46333 | 2.443 | 1.288558 | 1.16E-11 |
| PAS_chr1-4_0511 | Metalloprotease | 14.5 | 7.996667 | 0.481 | -1.05694 | 1.18E-11 |
| PAS_chr1-3_0201 | Hypothetical protein | 16.47333 | 38.29 | 2.045 | 1.032053 | 1.18E-11 |
| PAS_chr3_1074 | Hypothetical protein | 82.43 | 191.3033 | 2.144 | 1.100109 | 1.38E-11 |
| PAS_chr3_0349 | NADP(+)-dependent dehydrogenase | 69.61667 | 178.1933 | 2.302 | 1.202795 | 1.56E-11 |
| PAS_chr1-1_0072 | Fructose 1,6-bisphosphate aldolase, required for glycolysis and gluconeogenesis | 3165.197 | 1699.09 | 0.478 | -1.06447 | 1.56E-11 |
| PAS_chr4_0335 | Hypothetical protein | 13.3 | 32.14333 | 2.156 | 1.108625 | 1.68E-11 |
| PAS_chr1-4_0437 | Hypothetical protein | 2.67 | 7.896667 | 2.614 | 1.386195 | 1.80E-11 |
| PAS_chr2-1_0507 | Hypothetical protein | 3.656667 | 9.066667 | 2.177 | 1.122423 | 2.39E-11 |
| PAS_chr2-2_0333 | Putative kinase | 134.28 | 73.52 | 0.488 | -1.03445 | 3.97E-11 |
| PAS_chr1-4_0518 | Hypothetical protein | 13.78667 | 31.42667 | 2.019 | 1.0135 | 5.16E-11 |
| PAS_chr3_1075 | Hypothetical protein | 2.553333 | 7.806667 | 2.695 | 1.430235 | 9.62E-11 |
| PAS_chr4_0273 | Hypothetical protein | 15.70333 | 7.356667 | 0.415 | -1.26823 | 1.11E-10 |
| PAS_chr2-2_0323 | Co-chaperone that stimulates the ATPase activity of the HSP70 protein Ssc1p | 140.8233 | 80.35667 | 0.497 | -1.00797 | 6.21E-10 |
| PAS_chr3_0736 | Putative protein of unknown function | 4.486667 | 12.65333 | 2.49 | 1.31606 | 6.40E-10 |
| PAS_chr2-1_0074 | GTPase-activating protein for Sec4p and several other Rab GTPases, regulates exocytosis via its acti | 14.33333 | 7.196667 | 0.44 | -1.18567 | 6.82E-10 |
| PAS_chr1-3_0237 | Karyopherin/importin that interacts with the nuclear pore complex | 47.47333 | 25.99333 | 0.477 | -1.06914 | 7.09E-10 |
| PAS_chr3_0742 | Subunit of cleavage factor I (CFI) | 18.02667 | 40.6 | 2.012 | 1.008564 | 1.16E-09 |
| PAS_chr2-2_0406 | Hypothetical protein | 49.37 | 113.8367 | 2.056 | 1.039847 | 1.29E-09 |
| PAS_chr3_0403 | acetate--CoA ligase | 2.546667 | 7.853333 | 2.75 | 1.459186 | 1.54E-09 |
| PAS_chr3_0598 | Non-essential small GTPase of the Rho/Rac subfamily of Ras-like proteins | 71.42667 | 162.58 | 2.045 | 1.032161 | 1.70E-09 |
| PAS_chr1-4_0213 | Hypothetical protein | 100.7267 | 52.07667 | 0.45 | -1.15279 | 2.15E-09 |
| PAS_chr1-4_0621 | Major facilitator superfamily | 1.43 | 4.946667 | 3.063 | 1.614875 | 2.23E-09 |
| PAS_chr2-1_0037 | Nitrilase, member of the nitrilase branch of the nitrilase superfamily | 26.55667 | 63.36667 | 2.139 | 1.096838 | 2.52E-09 |
| PAS_chr3_0557 | Hypothetical protein | 3.766667 | 9.246667 | 2.178 | 1.12295 | 2.68E-09 |
| PAS_chr1-4_0581 | Hypothetical protein | 7.88 | 28.76 | 3.344 | 1.741695 | 3.92E-09 |
| PAS_chr4_0663 | Hypothetical protein | 16.22667 | 9.1 | 0.495 | -1.01352 | 5.24E-09 |
| PAS_chr3_0777 | Putative protein of unknown function | 6.173333 | 15.04667 | 2.154 | 1.106993 | 5.98E-09 |
| PAS_chr2-1_0252 | Putative protein of unknown function | 17.67 | 40.73 | 2.069 | 1.048652 | 6.41E-09 |
| PAS_chr2-1_0324 | Oligomeric mitochondrial matrix chaperone | 150.4733 | 77.61 | 0.449 | -1.15471 | 7.29E-09 |
| PAS_chr1-4_0258 | Hypothetical protein | 13.24667 | 31.36667 | 2.113 | 1.0796 | 9.97E-09 |
| PAS_chr4_0911 | hypothetical protein | 10.15333 | 23.89333 | 2.099 | 1.069662 | 1.09E-08 |
| PAS_chr3_0842 | Multifunctional protein with both hydroxymethylpyrimidine kinase and thiaminase activities | 22.53667 | 52.24667 | 2.044 | 1.031504 | 1.12E-08 |
| PAS_chr4_0807 | Hypothetical protein | 12.9 | 30.09 | 2.089 | 1.062981 | 1.26E-08 |
| PAS_chr1-4_0431 | Plasma membrane multidrug transporter of the major facilitator superfamily | 1.35 | 4.12 | 2.681 | 1.422692 | 1.71E-08 |
| PAS_chr3_0861 | Receptor for a factor receptor, transcribed in alpha cells and required for mating by alpha cells | 7.05 | 16.11333 | 2.022 | 1.015683 | 3.44E-08 |
| PAS_chr4_0961 | hypothetical protein | 0.93 | 3.066667 | 2.922 | 1.547001 | 4.22E-08 |
| PAS_chr2-1_0329 | Transcriptional activator of proline utilization genes | 14.88667 | 8.523333 | 0.499 | -1.00355 | 4.36E-08 |
| PAS_chr1-4_0369 | Hypothetical protein | 216.52 | 655.0867 | 2.912 | 1.542246 | 9.74E-08 |
| PAS_chr3_0590 | Homeodomain-containing transcriptional repressor of PTR2 | 23.96667 | 56.69667 | 2.105 | 1.073936 | 1.36E-07 |
| PAS_chr2-2_0223 | Hypothetical protein | 1.383333 | 3.813333 | 2.449 | 1.292074 | 1.42E-07 |
| PAS_chr2-1_0009 | Hypothetical protein | 3.62 | 9.376667 | 2.259 | 1.175747 | 1.44E-07 |
| PAS_chr4_0240 | Ferric reductase, reduces siderophore-bound iron prior to uptake by transporters | 2.816667 | 7.016667 | 2.193 | 1.133181 | 2.64E-07 |
| PAS_chr1-3_0170 | Zinc-finger DNA-binding protein | 8.403333 | 20.98 | 2.217 | 1.148523 | 2.77E-07 |
| PAS_chr2-2_0015 | One of several homologs of bacterial chaperone DnaJ, located in the ER lumen | 6.04 | 2.843333 | 0.412 | -1.27859 | 3.84E-07 |
| PAS_chr2-1_0870 | hypothetical protein | 2.886667 | 6.69 | 2.046 | 1.032495 | 3.95E-07 |
| PAS_chr2-2_0279 | Hypothetical protein | 38.54 | 88.58333 | 2.027 | 1.019554 | 4.14E-07 |
| PAS_chr2-1_0270 | Plasma membrane protein with roles in the uptake of protoprophyrin IX and the efflux of heme | 10.54 | 5.37 | 0.449 | -1.15573 | 5.27E-07 |
| PAS_chr1-3_0238 | Phosphatidylglycerolphosphate synthase, catalyzes the synthesis of phosphatidylglycerolphosphate | 5.616667 | 13.10667 | 2.042 | 1.029791 | 6.01E-07 |
| PAS_chr4_0065 | Protein involved in synthesis of the thiamine precursor hydroxymethylpyrimidine (HMP) | 6.77 | 17.04667 | 2.208 | 1.142707 | 8.14E-07 |
| PAS_chr4_0268 | Hypothetical protein | 1.98 | 6.806667 | 3.092 | 1.628722 | 1.36E-06 |
| PAS_chr3_0480 | Putative chaperone, homolog of E. coli DnaJ, closely related to Ydj1p | 12.14333 | 6.736667 | 0.486 | -1.03985 | 1.53E-06 |
| PAS_chr1-1_0413 | Hypothetical protein | 11.18 | 5.88 | 0.467 | -1.09737 | 1.66E-06 |
| PAS_chr1-4_0073 | Hypothetical protein | 25.48667 | 14.42 | 0.493 | -1.02013 | 2.26E-06 |
| PAS_chr2-1_0560 | Protein with similarity to human cystinosin, which is a H(+)-driven transporter | 2.996667 | 8.31 | 2.44 | 1.287044 | 4.72E-06 |
| PAS_chr4_0104 | Zinc-finger protein of unknown function | 2.706667 | 6.61 | 2.15 | 1.104435 | 9.18E-06 |
| PAS_chr1-4_0372 | hypothetical protein | 30.42333 | 16.69333 | 0.499 | -1.004 | 9.48E-06 |
| PAS_chr1-4_0110 | pterin-4-alpha-carbinolamine dehydratase | 61.01 | 26.07 | 0.4 | -1.32314 | 1.64E-05 |
| PAS_chr1-1_0328 | Hypothetical protein | 17.82 | 9.8 | 0.483 | -1.05075 | 1.67E-05 |
| PAS_chr2-1_0320 | Protein containing a UCS (UNC-45/CRO1/SHE4) domain | 3.85 | 2.183333 | 0.493 | -1.02061 | 0.000116 |
| PAS_chr1-1_0365 | Putative protein serine/threonine kinase expressed at the end of meiosis | 1.726667 | 4.126667 | 2.114 | 1.080168 | 0.000121 |
| PAS_chr3_0794 | Hypothetical protein | 2.446667 | 1.216667 | 0.44 | -1.18352 | 0.000127 |
| PAS_chr1-3_0041 | Serine/threonine protein kinase involved in activation of meiosis | 0.323333 | 1.126667 | 3.051 | 1.609164 | 0.000132 |
| PAS_chr3_0445 | Hypothetical protein | 6.823333 | 3.79 | 0.492 | -1.02238 | 0.000149 |
| PAS_chr1-3_0078 | S-adenosyl-L-methionine uroporphyrinogen III transmethylase | 1.673333 | 3.97 | 2.069 | 1.048915 | 0.000161 |
| PAS_chr4_0594 | Putative protein of unknown function | 1.323333 | 3.736667 | 2.483 | 1.31237 | 0.000201 |
| PAS_chr2-2_0262 | Hypothetical protein | 36.12333 | 78.46 | 2.044 | 1.031061 | 0.000399 |
| PAS_chr1-4_0408 | Amidase, removes the amide group from N-terminal asparagine and glutamine residues | 4.556667 | 2.57 | 0.495 | -1.01426 | 0.000735 |
| PAS_chr2-1_0655 | Nucleolar protein, component of the small subunit (SSU) processome | 0.993333 | 2.483333 | 2.206 | 1.141731 | 0.001033 |
| PAS_chr3_0288 | Hypothetical protein | 7.62 | 26.38 | 3.216 | 1.685458 | 0.001502 |
| PAS_chr2-1_0042 | Hsp70 (Ssa1p) nucleotide exchange factor, cytosolic homolog of Sil1p, which is the nucleotide exchan | 127.3567 | 55.86667 | 0.385 | -1.37809 | 0.00162 |
| PAS_chr1-4_0061 | hypothetical protein | 6.666667 | 3.713333 | 0.493 | -1.01938 | 0.001802 |
| PAS_chr3_0096 | Hypothetical protein | 0.696667 | 0.32 | 0.404 | -1.3089 | 0.002674 |
| PAS_chr3_0001 | Hypothetical protein | 3.333333 | 1.213333 | 0.324 | -1.62748 | 0.003442 |
| PAS_chr3_1225 | hypothetical protein | 0.593333 | 1.41 | 2.085 | 1.05991 | 0.004289 |
| PAS_chr1-3_0245 | Hypothetical protein | 2.453333 | 6.18 | 2.264 | 1.179182 | 0.006448 |
| PAS_FragB_0057 | Exoribonuclease II, mitochondrial | 0.343333 | 0.86 | 2.238 | 1.161892 | 0.006458 |
| PAS_chr3_1224 | hypothetical protein | 0.183333 | 0.546667 | 2.616 | 1.38761 | 0.015263 |
| PAS_chr3_0172 | Putative protein of unknown function | 0.443333 | 1.086667 | 2.182 | 1.125921 | 0.027443 |
| PAS_chr3_0196 | Hypothetical protein | 49.26667 | 21.19 | 0.422 | -1.24306 | 0.040037 |
| PAS_chr2-1_0134 | Hypothetical protein | 2.69 | 7.08 | 2.423 | 1.276702 | 0.044009 |
| PAS_chr2-2_0336 | Hypothetical protein | 4.803333 | 2.426667 | 0.458 | -1.12796 | 0.048518 |
