# Supplementary material for: Deciphering cell wall sensors enabling the construction of robust P. pastoris for single-cell protein production
Source: Biotechnol Biofuels Bioprod. 2023 Nov 17;16:178. doi: 10.1186/s13068-023-02428-7 (PMC10655344; doi:10.1186/s13068-023-02428-7)
Supplement: Supplementary file 2 — Additional file 2. Strains and plasmids used in this study. [file 13068_2023_2428_MOESM2_ESM.docx]

T**able S2 Strains and plasmids used in this study**

| **Strains and Plasmids** | **Relevant Characteristics** | **Sources** |
| --- | --- | --- |
| ***P. pastoris*** |  |  |
| X33 | Wild type, Mut^+^ | Lab stock |
| *△PAS_0305* | Zeocin | Lab stock |
| *△PAS_0305-cp* | Zeocin | Lab stock |
| ***E. coli*** |  |  |
| DH5α | F^-^, φ80*lacZ* Δ*M15*, Δ(*lacZYA-argF*)*U169*, *deoR*, *recA1*, *endA1*, *hsdR17*(rk^-^, mk^+^), *phoA*, *supE44*, *λ*^-^, *thi-1*, *gyrA96*, *relA1* | Lab stock |
| **Plasmids** |  |  |
| pPICZ-Cas9-gGUT1 | ori, Amp, Zeocin, T_DAS1_-Cas9-P_HTX1_-GUT1-gRNA2-T_AOX_ | Ref. 11 |
| pPICZ-Cas9-g*PAS_chr4_0305* | ori, Amp, Zeocin, T_DAS1_-Cas9-P_HTX1_-*PAS_chr4_0305*-gRNA-T_AOX_ | This study |
| pPICZ-Cas9-g*PAS_ chr2-1_0454* | ori, Amp, Zeocin, T_DAS1_-Cas9-P_HTX1_- *PAS_ chr2-1_0454*-gRNA-T_AOX_ | This study |
