# Supplementary material for: Deciphering cell wall sensors enabling the construction of robust P. pastoris for single-cell protein production
Source: Biotechnol Biofuels Bioprod. 2023 Nov 17;16:178. doi: 10.1186/s13068-023-02428-7 (PMC10655344; doi:10.1186/s13068-023-02428-7)
Supplement: Supplementary file 3 — Additional file 3. Primers used for strain and plasmid construction in this study. [file 13068_2023_2428_MOESM3_ESM.docx]

**Table S3. Primers used for strain and plasmid construction in this study**

| **Primer** | **Sequences (5′ 3′)** |
| --- | --- |
| *PAS_chr4_0305-up-F* | GAAGGTTCTAATACTAAGAACT |
| *PAS_chr4_0305-up-R* | AATTGGATTGAAGAGTACTAAT |
| *PAS_chr4_0305-down-F* | GCTGTACTATTAGTACTCTTCAATCCAATTAAGGGAGAAAAAGAAAAGTTAT |
| *PAS_chr4_0305-down-R* | ACAATTCAGATATTCGACGGTT |
| *gPAS_chr4_0305-1-F* | GGATGACTGATGAGTCCGTGAGGACGAAACGAGTAAGCTCGTCTCATCCTATTCTAATACATTGTTTTAGAGCTAGAAATAGCA |
| *gPAS_chr4_0305-1-R* | ACGGGAAGTCTTTACAGTTT |
| *gPAS_chr4_0305-2-F* | CTCCTAACTAAAACTGTAAAGACTTCCCGTTTAAACTTTTCTTTTCTTCT |
| *gPAS_chr4_0305-2-R* | GTTTCGTCCTCACGGACTCATCAGTCATCCTTTGATTTGTTTAGGTAACT |
| *PAS_chr2-1_0454-up-F* | GAACTCTCAGTCCCTCTTGGCCTAC |
| *PAS_chr2-1_0454-up-R* | CCCCGTTGTGCAATAAAGCTAAGGA |
| *PAS_chr2-1_0454-down-F* | CTGCTTCCTTAGCTTTATTGCACAACGGGGGTTTCTCCAATCAATGAAAGCATTA |
| *PAS_chr2-1_0454-down-R* | GTTGCACGCTCTTCTCCAGCTAGCT |
| *gPAS_chr2-1_0454-1-F* | TACGAACTGATGAGTCCGTGAGGACGAAACGAGTAAGCTCGTCTTCGTATACCAATTGGCTACGTTTTAGAGCTAGAAATAGCA |
| *gPAS_chr2-1_0454-1-R* | ACGGGAAGTCTTTACAGTTT |
| *gPAS_chr2-1_0454-2-F* | CTCCTAACTAAAACTGTAAAGACTTCCCGTTTAAACTTTTCTTTTCTTCT |
| *gPAS_chr2-1_0454-2-R* | GTTTCGTCCTCACGGACTCATCAGTTCGTATTTGATTTGTTTAGGTAACT |
